# Prevalences of Anorexia, Autism, and Schizophrenia, Are Strongly Associated With Average Annual Temperatures: Systematic Review and Linear Regression Analysis

**DOI:** 10.1002/brb3.70999

**Published:** 2025-10-20

**Authors:** Sofia Philippou, Konstantinos Voskarides, Andreas Chatzittofis

**Affiliations:** ^1^ Medical School University of Cyprus Nicosia Cyprus; ^2^ Department of Basic and Clinical Sciences University of Nicosia Medical School Nicosia Cyprus; ^3^ School of Veterinary Medicine University of Nicosia Nicosia Cyprus; ^4^ Department of Clinical Sciences/Psychiatry Umeå University Umeå Sweden

**Keywords:** cold, geographical pattern, latitude, psychosis

## Abstract

**Background:**

The impact of potential environmental influences, like temperature changes and latitudinal gradient, has not been investigated in depth in psychiatric diseases. The aim of this project was to investigate the association of geographical latitude and temperature with the prevalence of psychiatric disorders.

**Methods:**

Linear regression analysis was performed for 201 countries by analyzing average annual temperatures and age‐standardized rates (prevalence) of seven major psychiatric entities. A systematic review was also performed, investigating if these correlation data were supported by published original studies.

**Results:**

Linear regression analysis showed a significant correlation between average annual temperatures and age‐standardized rates (*p* < 0.0001) for three psychiatric disorders: anorexia, autism, and schizophrenia. Systematic review analysis showed that the prevalence of autism and schizophrenia is potentially influenced by geographic and climatic factors. However, no published data were identified to support the findings for anorexia.

**Conclusion:**

These preliminary findings underscore the complexity of interactions between environmental, genetic, and socioeconomic factors for psychiatric diseases. The association between temperature and prevalence of psychiatric diseases needs further investigation to reveal any unknown epidemiological factors that contribute to disease pathogenesis.

## Graphical abstract

Psychiatric disease prevalence and the average annual temperatures of 201 countries. A strong association is observed between temperature and disease prevalence for anorexia, autism, and schizophrenia.

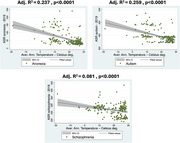

## 1 Introduction

Geographical latitude significantly influences the prevalence and distribution of autoimmune, neurological, and psychiatric diseases. For instance, diseases like multiple sclerosis (MS) are more common in higher latitudes. Additionally, colder climates can exacerbate symptoms of conditions like rheumatoid arthritis and systemic lupus erythematosus. The effect of latitude in patients of MS has widely been demonstrated, revealing that the prevalence increases proportionally with the latitude (Simpson et al. 2019). This has also been demonstrated in studies investigating the impact of latitude on depression, with similar results (increased prevalence along increased latitude) (Patten et al. 2017).

Schizophrenia, with a worldwide prevalence among adults being around 1% (World Health Organization [WHO] 2022), is a psychiatric disorder with complex pathophysiology, including genetic and environmental factors. A number of neurobiological systems are implicated in the pathogenesis of this disease, including the dopaminergic, the glutamatergic, and the GABAergic systems and inflammatory‐immune dysregulation (Lang et al. 2007) (Maia and Frank 2017) (Zohreh and Sheng 2023) (L. J. Cui et al. 2024). Autism spectrum disorder (ASD) is a developmental disorder whose pathogenetic mechanism is unknown, implicating both genetic and non‐genetic factors (Sauer et al. 2021). ASD has a strong genetic background, and possible biomarkers include cytokines, growth factors, measures of oxidative stress, vitamin D, neurotransmitters, and hormones (Parellada et al. 2023).

The association between latitude and psychiatric diseases has been previously explored, including investigation of the possible mechanisms. An epidemiological study reported a significant positive correlation between latitude and the incidence of schizophrenia in males (Saha et al. 2006). One potential mechanism regarding the prevalence of schizophrenia and autism might be vitamin D deficiency (Syed et al. 2017). Similarly, low levels of ultraviolet B exposure, particularly in northern countries, have been associated with increased risk of autism. Regarding other psychiatric disorders, a systematic review reported that patients with anorexia nervosa had lower vitamin D levels, even when dietary vitamin D uptake was like the healthy controls (Veronese et al. 2015).

There is a significant gap in the literature examining the relationship of temperature/geographical latitude and psychiatric disorders, particularly schizophrenia, ASD, and anorexia nervosa. Further research is needed to gain a more detailed understanding of those diseases.

The aim of this study was to address and assess the possible relationship between the prevalence of psychiatric disorders and annual average temperature worldwide through linear regression analysis. Additionally, we conducted a systematic review to examine literature evidence for the connection between temperature and prevalence of psychiatric disorders.

## 2 Methods

### 2.1 Temperature and Prevalence Data

Average annual temperature (AAT) by country (years 1991–2020) is based on gridded climatologies from the ClimateChangeKnowledgePortal. Age‐standardized rates (ASR) (prevalence) of psychiatric disorders were adopted by the Global Burden of Disease Study (Institute for Health Metrics and Evaluation 2021), data of 2019. Temperature and prevalence data can be found in Table S1.

### 2.2 Statistical Analysis

Statistical analysis was performed by the statistical package STATAv.13 (StataCorp LLC, Texas, USA). A linear regression analysis was performed for AAT and prevalence (ASR) of psychiatric disorders (depression, dysthymia, bipolar disorder, anxiety, anorexia, autism, and schizophrenia). Significant level alpha was set to 0.01.

### 2.3 Systematic Review Method

We conducted a systematic literature review to identify the association between the prevalence of specific mental disorders and geographic latitude as per PRISMA guidelines. We included all related studies published in English, and we excluded studies with < 10 patients, systematic and narrative review articles, abstracts, case reports, and case series.

The search was performed across Medline/PubMed, Scopus, and Embase from their inception until September 2024. We included the following search terms: “Geographic latitude,” “North‐south incidence gradient,” “Geographic variation,” “Geographic location,” “Latitudinal gradient,” “schizophrenia,” “anorexia,” and “autism.”

**FIGURE 1:**
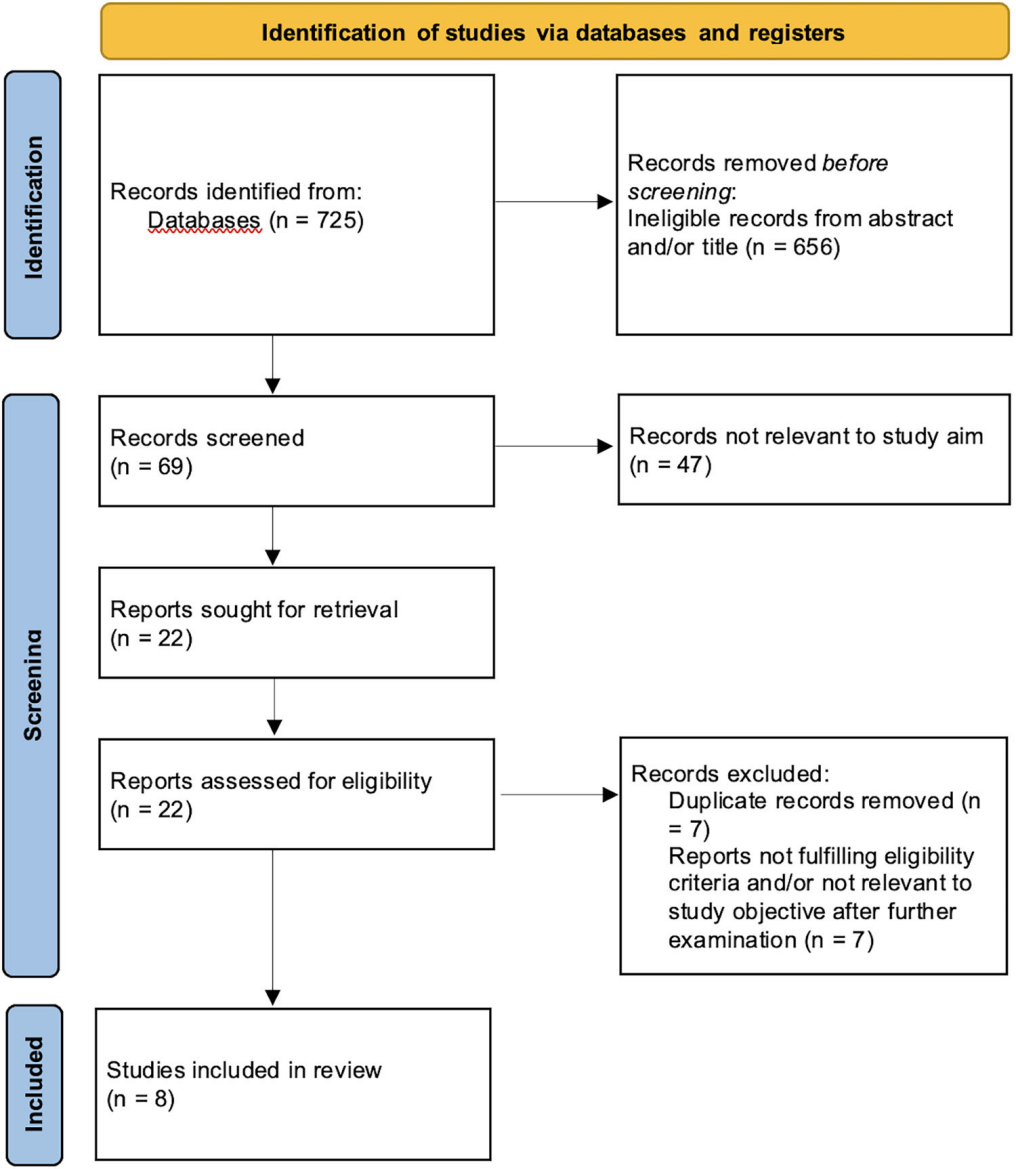
A PRISMA chart describing the inclusion/exclusion process of the systematic review analysis.

A total of 725 studies were identified, of which 69 were screened to assess their eligibility. After excluding systematic reviews and studies including < 10 patients, we identified 22 studies that met our eligibility criteria. After further examination of the full articles and removing duplicates, we included eight studies (Figure 1).

## 3 Results

### 3.1 AATs and Psychiatric Disease Prevalence

**FIGURE 2:**
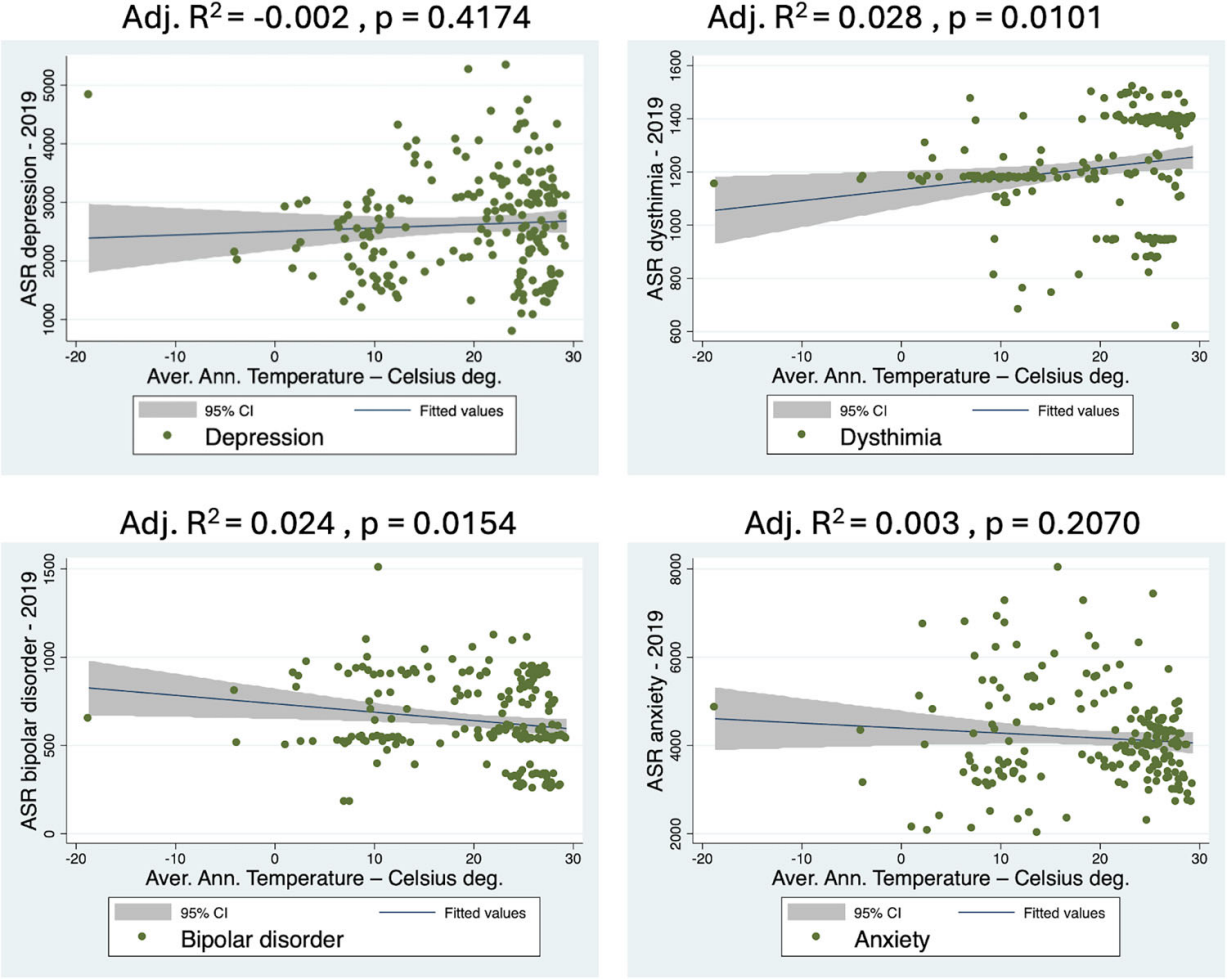
Linear regression analysis showing the psychiatric disease prevalence and the average annual temperatures of 201 countries for four diseases with no statistically significant association.

**FIGURE 3:**
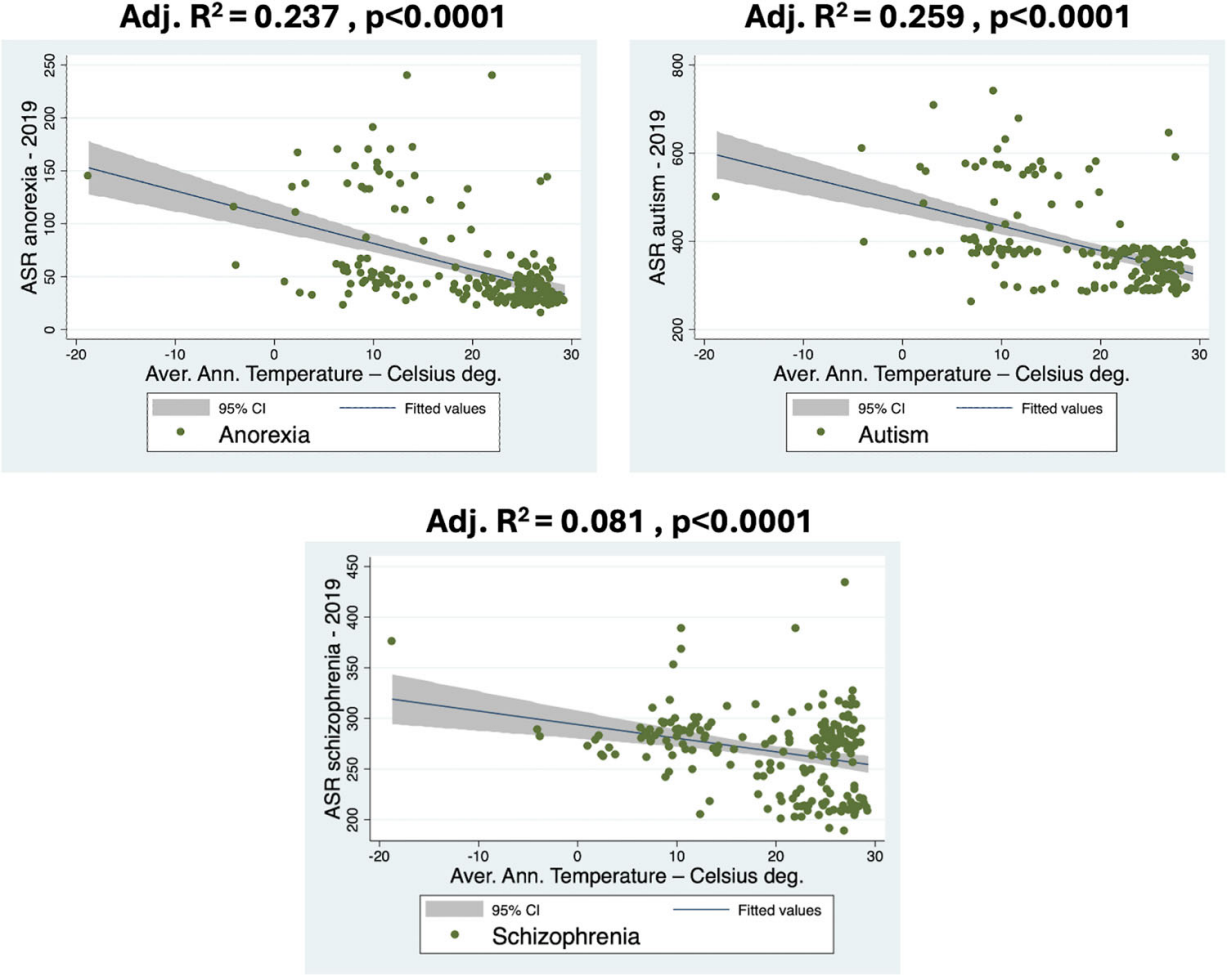
Linear regression analysis showing the psychiatric disease prevalence and the average annual temperatures of 201 countries for three diseases with highly significant association.

In order to investigate the relationship between prevalence of mental diseases and AAT, a linear regression analysis was performed using AAT and age‐ASR. Scatter plots illustrate the relationship between AAT and ASR of mental disorders (Figures 2 and 3). AAT and ASR values are inversely associated, indicating decreasing prevalence along higher temperatures. Statistical significance was found for anorexia (adj. *R* ^2^ = 0.237, *p* < 0.0001), schizophrenia (adj. *R* ^2^ = 0.081 *p* < 0.0001) and ASD (adj. *R* ^2^ = 0.259, *p* < 0.0001). Moreover, in these three psychiatric disorders, most of the cases are at the right corner of the diagram, showing that as the temperature increases, the prevalence rates are lower. These findings suggest that lower temperatures are related to a higher prevalence of anorexia, schizophrenia, and ASD.

### 3.2 Systematic Review Analysis

In this review, eight studies met the eligibility criteria (included in Table 1). The process is described in the flow diagram (Figure 1).

**TABLE 1:** Characteristics of selected studies.

Study first author	Publication year	Country	Psychiatric disease	Age	Number of patients
Kate Hoffman (Hoffman et al. 2012)	2012	USA	Autism	8 years old	532
Zoe E Reed (Reed et al. 2021)	2021	Sweden, UK	Autism	9–12 years old	16,667 and 11,594
Kate Hoffman (Hoffman et al. 2017)	2017	USA	Autism	6 years old	13,507
Loretta Thomaidis (Thomaidis et al. 2020)	2020	Greece	Autism	10 and 11 years old	182,879
Jonna Perälä (Perälä et al. 2008)	2008	Finland	Schizophrenia	>= 30 years old	8028
Roberto Amato (Amato et al. 2010)	2010	Sub‐Saharan Africa, North Africa, Europe, the Middle East, South/Central Asia, East Asia, Oceania, and the Americas	Schizophrenia	n/a	938
Andrew Shaner (Shaner et al. 2007)	2007	Russia	Schizophrenia	n/a	1431
S Saha (Saha et al. 2006)	2006	27 countries	Schizophrenia	n/a	n/a

The quality of prospective and retrospective cohort studies, along with case‐control studies, was evaluated using the Newcastle‐Ottawa Scale (NOS). For details see Table S2.

Regarding schizophrenia, four papers met our inclusion criteria. Perälä et al. (2008) investigated the incidence of psychotic disorders in Finland, reporting that schizophrenia and other nonaffective psychoses, but not affective psychoses, were more prevalent in the North (OR = 7.72) and in the East (OR = 3.99). Furthermore, a study of 938 patients evaluated the genetic aspect in schizophrenia and vitamin D, suggesting a latitude‐driven adaptation for both schizophrenia and vitamin D‐related genes. Specifically, nine latitude‐related genes are related to both vitamin D and schizophrenia. For example, expression of the *NTRK3* gene is reduced among patients with schizophrenia. Moreover, reduced expression of *SMARCA2* associated with a specific genetic variant may contribute to the increased risk of schizophrenia (Amato et al. 2010). Furthermore, a study reported that geographic latitude may be related with the age of onset of schizophrenia, showing a linear latitudinal gradient in the mean age at onset of schizophrenia in 13 Northern Hemisphere cities (Shaner et al. 2007). Finally, a comparative study showed that the incidence of schizophrenia increases proportionally with latitude, a multivariate association including vitamin D deficiency (Saha et al. 2006).

Regarding autism, a study conducted in the United States revealed that children living in specific urban areas (e.g., Alamance, Durham, and Orange Counties) showed higher prevalence of the disease and additionally were also 1.10–1.27 times more likely to be diagnosed by the age of 8 (Hoffman et al. 2012). However, this effect was attenuated by known individual‐level confounders. Moreover, it seems that there is a geographical variation in the prevalence of autistic traits with variation in both genetic and environmental influences, as investigated in Sweden and the UK. In Sweden, genetic influences are higher in the south and the center of the country, while environmental influences are greatest in the south and north, with reduced environmental influence across the central band. In the UK, genetic influences are greater in the south, the Midlands, and the north of England, while environmental influences are greatest in the south and east of the UK (Reed et al. 2021). In a 2017 study, a study investigating geographic patterns of ASD in the United States reported a higher possibility of diagnosing autism in children born in New England and Indiana and decreased odds in southern and central regions (Hoffman et al. 2017). Interestingly, these patterns were not explained by common confounders such as maternal age, birth year, child's sex, community income, or prenatal exposure to hazardous air pollutants. In a 2020 Greek study, the authors showed that the more northern regions as well as the Attica region had higher prevalence of autism. The authors attribute the variance to differences in diagnostic procedures, availability of services, and mental health workers with experience in ASD (Thomaidis et al. 2020).

No published studies data were identified for anorexia nervosa.

## 4 Discussion

In this study, we report that lower temperatures are linked to increased prevalence of schizophrenia, ASD, and anorexia nervosa. The effects of latitude and temperature on the prevalence of psychiatric disorders have not been fully investigated in the literature so far. However, previous preliminary results, especially for schizophrenia and ASD, support our findings (Saha et al. 2006) (Syed et al. 2017). Nevertheless, a definitive link between the two, as well as the underlying mechanism, has yet to be determined. Additionally, it must be mentioned that *R* ^2^ for schizophrenia is quite small (adj. *R* ^2^ = 0.081), showing that this association is not as strong as in autism and anorexia (Figure 3).

Latitude is related to a variety of factors, such as genetic variation in populations, biometeorological factors such as temperature and ultraviolet radiation, infectious agents, and socioeconomic factors. In fact, more developed countries are clustering within higher latitude bands, and there are differences in urbanicity across latitude gradients.

Regarding the incidence of autism, a north‐south gradient was noted. Specifically, the southern parts of Greece and some southern US states exhibited lower prevalence of autism. A possible hypothesis would be the involvement of vitamin D. In fact, a review study showed that vitamin D deficiency may also play a crucial role in the development of the disease, as the prevalence increases along with the latitude, whereas it decreases near the equator (Syed et al. 2017). A 2021 systematic review investigating the worldwide prevalence of autism showed that autism had a high prevalence in Asian countries, including South Korea and Japan (Santomauro et al. 2021). The dysregulation of the immune system might be a possible mechanism for this association, as it has already been reported in psychiatric disorders, including ASD and schizophrenia (Parellada et al. 2023) (Erbescu et al. 2022).

Indeed, findings regarding the neurobiology of ASD include dysregulation of cytokines, growth factors, measures of oxidative stress, neurotransmitters, hormones, and vitamin D deficiency. Especially regarding cytokines, including IL‐10, meta‐regression analyses suggest interaction with latitude in patients with ASD (Saghazadeh et al. 2019). Also, vitamin D is implicated in fetal brain development and is linked to the latitude of the birthplace of the child's parents (Botsas et al. 2023). Vitamin D deficiency in the early years of life may be associated with neuronal changes, increasing the risk of psychiatric diseases such as autism and schizophrenia (Eyles et al. 2013). Another possible contributing factor is the temperature (Zhou et al. 2024). Specifically, as temperature increases, especially with global warming, it can lead to epigenetic changes, which might affect the offspring.

For schizophrenia, the most relevant study reported that the incidence increases proportionally with latitude, and this difference is multivariate, including vitamin D deficiency (Saha et al. 2006). Additionally, the authors showed that people living in Norway (natives and immigrants) with low vitamin D levels exhibited higher risk for psychotic disorders (Andreassen et al. 2010). Systematic reviews and meta‐analyses of observational studies investigating serum vitamin D levels in schizophrenia revealed a strong association between vitamin D deficiency and schizophrenia (Zhu et al. 2020). More than 60% of schizophrenic patients had vitamin D deficiency, and vitamin D‐deficient persons were more than two times more likely to have schizophrenia compared to those with vitamin D sufficiency (Valipour et al. 2014) (X. Cui et al. 2021). In fact, vitamin D deficiency during development was proposed as a risk factor for schizophrenia (Kesby et al. 2011) (J. McGrath et al. 2003) (J. J. McGrath et al. 2010). Developmental vitamin D deficiency alters dopamine ontogeny and interacts with other risk factors for schizophrenia, such as the immune system and the function of the placenta. It can influence postnatal behavior and genomic and epigenomic mechanisms (Kesby et al. 2011) (J. McGrath et al. 2003) (J. J. McGrath et al. 2010). Immunological dysregulation has been implicated in the pathophysiology of schizophrenia with an increase of the pro‐inflammatory cytkine levels, including interleukin‐6 (IL‐6). Vitamin D is a modulator of the immune system, and higher IL‐6 and C‐reactive protein levels were reported in patients with psychosis compared to controls. Levels of IL‐6 showed a negative correlation with vitamin D in the psychosis group, suggesting a mediating role of vitamin D in psychosis risk (Delaney et al. 2019). Another possible correlation is related to vitamin D and genes associated with vitamin D. For example, *ZMIZ1* gene, which is responsive to vitamin D, was marked as a risk factor for some latitude‐risk diseases (Parnell et al. 2018).

Additionally, disruption of circadian rhythms is commonly observed in psychiatric diseases, including schizophrenia and autism. Through time, human populations adapted at different latitudes by genetic adaptation of their circadian clock systems. This adaptation involves genetic risk variants associated with neuropsychiatric conditions such as schizophrenia (Forni et al. 2014).

The absence of published research on the association of temperature and the prevalence of anorexia nervosa makes the findings of this study less robust compared to schizophrenia and autism and needs replication. If these findings are replicated, a possible hypothesis would be the involvement of vitamin D as proposed in other psychiatric disorders. However, studies that did not meet our inclusion criteria for anorexia have shown that vitamin D deficiency is associated with increased anorexia prevalence (Veronese et al. 2015). Finally, a recent hypothesis was published on sun exposure and anorexia nervosa, proposing a potential environmental–biological interaction (Phillipou 2025). This hypothesis urges for the investigation of environmental factors such as sun exposure and the effects of UV radiation in anorexia nervosa. This hypothesis proposes even mechanisms independently of vitamin D, such as effects on neurocognitive function and flexible thinking styles, as well as neurotransmitter systems and neuroendocrine functions. Another exploratory study using the distribution of bibliographic references in scientific literature on anorexia nervosa reported a similar pattern as for disorders that are associated with high latitudes (Vazquez et al. 2006). Besides the important limitations of that study, the authors also proposed a possible link between prevalence of anorexia and latitude.

Importantly, limitations of our study need to be addressed. First, in the systematic review part of our study, due to the lack of data in the literature, only eight studies met our inclusion criteria. Moreover, we did not find published data for anorexia, meeting the inclusion criteria. Hence, data may not be easily generalized. It is important to acknowledge that several confounding factors may have influenced the findings of this study. We did not control for socioeconomic variables such as each country's median annual income, gross domestic product (GDP), or degree of industrialization. Socioeconomic deprivation and sociocultural influences are known to be associated with psychiatric disorders, indicating that industrialization and related lifestyle changes could affect disease risk. Additionally, using prevalence studies from different regions, with differences in evaluation methods, structure, and capacity of healthcare systems, is challenging. These variations in healthcare infrastructure and diagnostic capabilities may impact the reported prevalence of psychiatric conditions. Previous research has shown that global disparities and gaps in access to healthcare contribute to significant differences in the diagnosis and treatment of psychiatric disorders—leading to underreporting in resource‐limited regions and potential detection bias in more developed healthcare systems. While our analysis emphasizes AAT, other environmental factors, such as air pollution, may also significantly influence the prevalence of psychiatric conditions. Elements like chronic stress, dietary habits, exposure to pollutants, and seasonal affective disorder (SAD) may further contribute to regional differences in mental health outcomes.

Further, genetic predisposition significantly influences the development of psychiatric diseases, with certain genetic variants increasing susceptibility to these conditions. These genetic factors can confound studies examining environmental influences on psychiatric disease prevalence.

## 5 Conclusion

In conclusion, this study focuses on the effect of temperature in three different mental disorders: schizophrenia, autism, and anorexia. This was expanded further through a systematic review analysis. The effect of variation was especially remarked for schizophrenia and autism, whereas anorexia findings were not supported by published data. Thus, the lack of supportive literature calls for caution when interpreting these results. Further studies are needed to further explore the effect of latitude and temperature on psychiatric disorders, as well as the possible mechanisms. Further knowledge on this topic will aid in the prevention and management of the potential factors that influence these diseases (e.g., low vitamin D levels). Moreover, this will advance genetic, epigenetic and epidemiological research, detecting specific population groups at risk.

## Author Contributions

**Sofia Philippou**: writing – original draft, writing – review & editing, data curation, methodology. **Konstantinos Voskarides**: writing – original draft, writing – review & editing, conceptualization, methodology‐statistical analysis. **Andreas Chatzittofis**: writing – original draft, writing – review & editing, conceptualization, methodology, supervision.

## Peer Review

The peer review history for this article is available at https://publons.com/publon/10.1002/brb3.70999.

## Supporting information



Average annual temperatures (1990‐2020) and age‐standardized rates (1999) of psychiatric diseases.

Quality evaluation of included studies using of the Newcastle‐Ottawa scale.

## Data Availability

The original contributions presented in the study are included in the article/ Supplementary Material. Further inquiries can be directed to the corresponding author.
