# Supplementary material for: Prevalences of Anorexia, Autism, and Schizophrenia, Are Strongly Associated With Average Annual Temperatures: Systematic Review and Linear Regression Analysis
Source: Brain Behav. 2025 Oct 20;15(10):e70999. doi: 10.1002/brb3.70999 (PMC12537842; doi:10.1002/brb3.70999)
Supplement: Supplementary file 2 — Quality evaluation of included studies using of the Newcastle‐Ottawa scale. [file BRB3-15-e70999-s002.docx]

**Evaluation of the quality of included studies by the use of the Newcastle-Ottawa scale.**

| **Study** | **Study design** | **Selection (up to 4)** | **Comparability (up to 2)** | **Outcome (up to 3)** | **Total** |
| --- | --- | --- | --- | --- | --- |
| Hoffman K. et al [18] | Cross-sectional | 3 | 2 | 3 | 8/9 |
| Reed Z.E. et al  [19] | Retrospective | 2 | 2 | 3 | 7/9 |
| Hoffman K. et al  [20] | Prospective | 2 | 2 | 3 | 7/9 |
| Thomaidis L.  [21] | Prospective | 3 | 1 | 3 | 7/9 |
| Perälä J. et al  [15] | Retrospective | 3 | 2 | 1 | 6/9 |
| Amato R. et al  [16] | Prospective | 2 | 2 | 2 | 6/9 |
| Shaner A. et al  [17] | Retrospective | 2 | 2 | 2 | 6/9 |
| Saha S. et al  [10] | Comparative | 2 | 1 | 2 | 5/9 |

The quality of prospective and retrospective cohort studies, along with case-control studies, was evaluated using the Newcastle-Ottawa Scale (NOS).

The NOS evaluates studies in three domains: Selection (up to 4 points), Comparability (up to two points) and Outcome (up to 3 points). A maximum of 9 points can be given. In this study, a score of at least 7 is considered high quality, 5-6 as moderate, and 4 or lower low quality.
